# A cell type transcriptome atlas for human pituitary, adrenal, thyroid and minor salivary glands

**DOI:** 10.1186/s12864-026-13514-z

**Published:** 2026-11-05

**Authors:** Philip Dusart, Eike Struck, Martin Zwahlen, Kalle von Feilitzen, Fredrik Pontén, Cecilia Lindskog, Jacob Odeberg, Mathias Uhlén, Lynn Marie Butler

**Affiliations:** 1 Department of Protein Science, SciLifeLab, KTH Royal Institute of Technology, 171 21 Stockholm, Sweden; 2 Translational Vascular Research, Department of Clinical Medicine, The Arctic University of Norway, 9019 Tromsø, Norway; 3 Department of Immunology, Genetics and Pathology, Uppsala University, 752 37 Uppsala, Sweden; 4 Coagulation Unit, Department of Hematology, Karolinska University Hospital, 171 76 Stockholm, Sweden; 5 The University Hospital of North Norway (UNN), 9019 Tromsø, Norway; 6 Clinical Chemistry and Blood Coagulation Research, Department of Molecular Medicine and Surgery, Karolinska Institute, 171 76, Stockholm, Sweden; 7 Clinical Chemistry, Karolinska University Laboratory, Karolinska University Hospital, 171 76 Stockholm, Sweden

**Keywords:** Cell profiling, Bulk RNA-seq, Gene expression specificity, Adrenal, Pituitary, Salivary, thyroid

## Abstract

The human pituitary, adrenal, thyroid and minor salivary glands have distinct yet interconnected roles in endocrine and exocrine regulation, contributing to systemic homeostasis, metabolism, stress response, and oral health. Despite their importance, transcriptional characterisation at the single-cell level remains relatively limited for these glands, compared to other tissues. Genes with cell type specific expression typically encode proteins essential for specialised functions, making their identification key for understanding physiology and pathophysiology. Single-cell RNA sequencing (scRNA-seq) has facilitated the profiling of such genes, but challenges such as dissociation-induced artefacts, protocol-dependent cell loss, and data sparsity limit the analysis of certain cell types and lowly expressed genes. Here, we applied an integrative network analysis to a dataset of over 1400 bulk RNA-seq samples from human pituitary, adrenal, thyroid, and minor salivary glands to predict genome-wide, cell type-enriched transcriptomes. Cross-gland analyses revealed core transcriptional similarities among functionally related cell types, such as neuroendocrine cells, while tissue subset analyses revealed pituitary gonadotropes as the predominant site of sex-specific variation. This study provides a useful additional resource for investigating glandular function in health and disease.

**Supplementary information:**

The online version contains supplementary material available at https://doi.org/10.1186/s12864-026-13514-z.

## Introduction

The human pituitary, adrenal, thyroid and minor salivary glands exhibit both shared and distinct characteristics. The pituitary, adrenal and thyroid glands are primarily endocrine, secreting hormones directly into the bloodstream to regulate systemic functions. In contrast, the minor salivary glands are exocrine, releasing enzymes and mucus into the oral cavity [1, 2]. The pituitary gland acts as a central regulator of other endocrine organs, influencing thyroid and adrenal activity via hypothalamic–pituitary feedback loops [2, 3]. The adrenal glands mediate stress responses and regulate electrolyte balance through cortisol, aldosterone, and catecholamine secretion [2, 4], while the thyroid primarily regulates metabolism [2, 5]. The minor salivary glands, distributed throughout the oral cavity, produce saliva containing amylase, lipase, and mucous secretions, supporting digestion and oral health [1]. Although each gland has specialized functions, they operate within integrated homeostatic networks, collectively regulating metabolism, growth, stress responses and immune defence [2].

Deciphering glandular function in health and disease requires an understanding of the transcriptional landscape of their constituent cell types. Compared with many other tissues, human pituitary, adrenal, thyroid and minor salivary glands tend to be underrepresented across large-scale scRNA-seq databases, such as Tabula Sapiens [6] and CZ CELLxGENE [7], and are often only found as a limited number of control samples in studies of tumorous or diseased glandular tissue. The use of scRNA-seq comes with specific challenges - the dissociation of tissue can induce transcriptional artefacts, both from loss of the native microenvironment [8] and associated stress responses [9, 10], and some genes can be underrepresented [11]. Certain cell types are sensitive to extraction protocols [9], whilst others require extensive, damaging proteolytic digestion to isolate [12, 13]. Compared to bulk RNA-seq, scRNA-seq yields less stable and more variable data, with a high number of zero values, particularly for lowly expressed genes [14–17]. Computational techniques can partially address this dropout rate [18, 19], but the methods for doing so remain contentious [20]. Most scRNA-seq studies typically process samples from a limited number of donors, increasing the risk of underestimating biological variability and inflating false positives in differential expression analyses [21–23].

Here, we generate a genome-wide, cell type-enriched transcriptome atlas for human pituitary, adrenal, thyroid, and minor salivary glands, by applying our previously developed integrative correlation analysis [24–26] to bulk RNA-seq datasets. This approach circumvents key limitations of scRNA-seq: no prior cell isolation is required, hundreds of samples are analysed together to mitigate biological variability, lowly expressed cell enriched transcripts can be identified and it enables well powered group comparisons [26, 27]. Cross-gland analyses revealed shared gene enrichment between functionally related cell types, such as adrenal medulla cells and other endocrine secretory populations. Sex-stratified analyses identified pituitary gonadotropes as the cell type with the most marked male–female transcriptional differences. All data is available in the Single Cell Resource section on the Human Protein Atlas (HPA) website (www.proteinatlas.org/humanproteome/single+cell/tissue+cell+type).

## Results

### Identification of cell type transcriptome profiles in human pituitary, adrenal, thyroid and minor salivary glands

#### Cell type reference transcripts correlate across unfractionated RNA-seq data

**Fig. 1:**
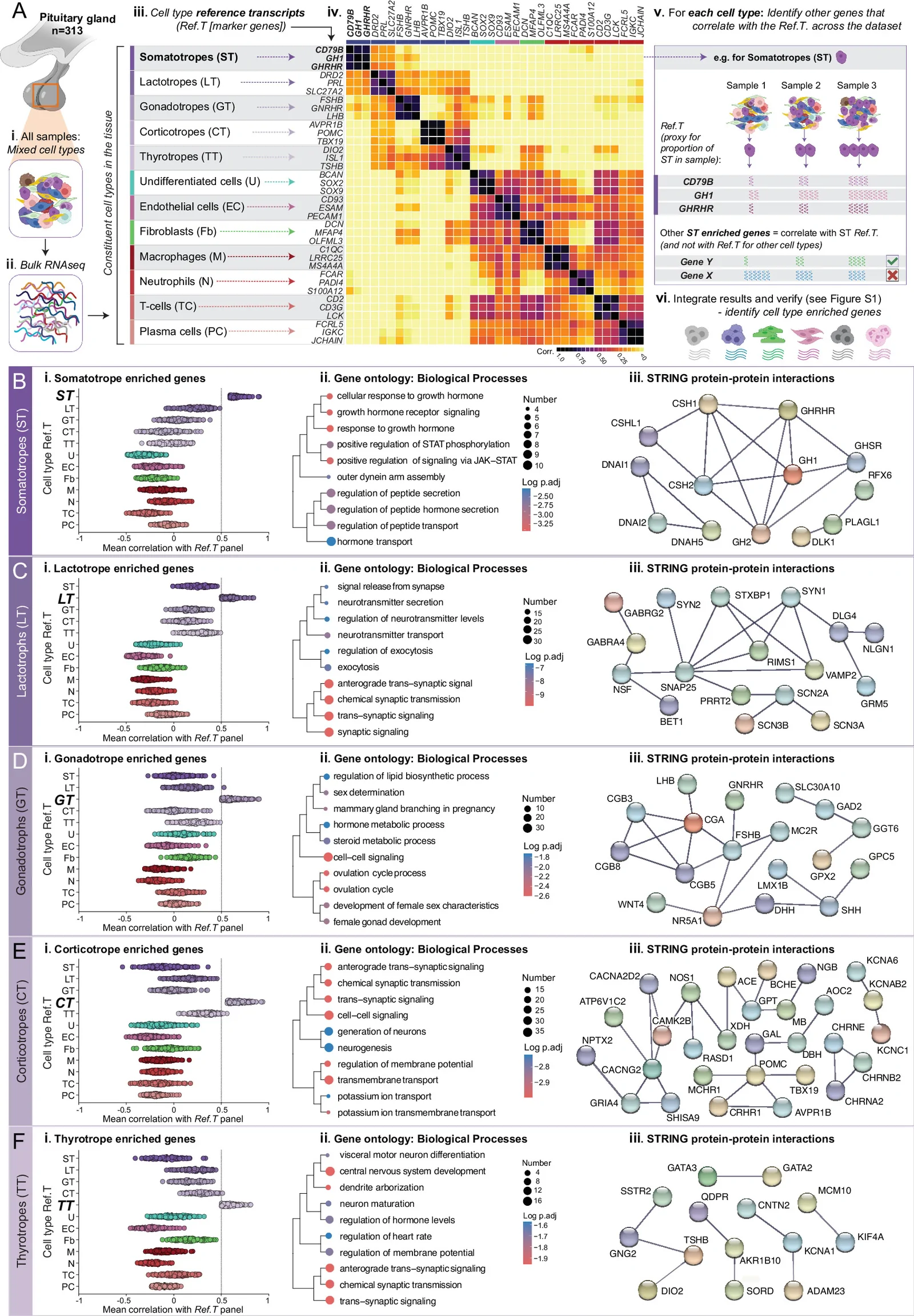
Integrative co-expression analysis of unfractionated human pituitary gland tissue RNA-seq can resolve constituent cell type enriched genes. **A** Bulk RNA-seq datasets for human pituitary gland were retrieved from GTEx V10 (*n* = 313) (**i**) each sample contained mixed cell types, contributing (**ii**) differing proportions of mRNA to each sequenced sample. To profile cell type-enriched transcriptomes constituent cell types for each tissue were identified and for each (**iii**) reference transcripts (*Ref.T*.) for ´virtual tagging´ were selected, primarily based on predicted cell specificity from in house protein profiling and/or existing literature. (**iv**) Matrix of correlation coefficient values between selected *Ref.T*. across the sample set. Schematic representation of the analysis used to predict cell type enriched genes, based on (**v**) identification of genes with similar expression patterns to the corresponding selected *Ref.T* and (**vi**) integration of the data across cell types. See also Figure S1 for more detailed explanation. For the predicted cell type enriched genes in (**B**) somatotropes [ST], (**C**) lactotropes [LT], (**D**) gonadotropes [GT], (**E**) corticotropes [CT] and (**F**) thyrotropes [TT], plots show (i) Mean correlation coefficients vs. all *Ref.T*. panels, (ii) significant gene ontology (GO) terms and (iii) evidence of interaction between the encoded proteins. See also Figure S1 and Table S1 Tab B

To identify cell type-enriched transcriptome profiles in human pituitary, adrenal, thyroid, and minor salivary glands we conducted an analysis based on a modified version of our previously published method [24–27]. Bulk RNA-seq data was obtained from the V10 Genotype-Tissue Expression (GTEx) portal [28] (https://gtexportal.org) from human pituitary gland (*n* = 313), adrenal gland, (*n* = 295), thyroid (*n* = 684), or minor salivary gland (*n* = 181). Samples from the same tissue type were analysed together (termed a ´*sample set*´); see Fig. 1 for method overview, using pituitary gland as an example. As each sample was unfractionated, it contained a mix of the constituent cell types found within the tissue (Fig. 1A.i), which thus contributed differing proportions of mRNA transcripts measured by RNA-seq (Fig. 1Aii). For each sample set, candidate cell type specific marker genes (termed ´reference transcripts´ [*Ref.T*.]) were selected for each of the major constituent cell types (Fig. 1A.iii), based on: in-house proteomic profiling [29], older ´non-omics´ descriptions of cell type specific genes [30, 31], scRNA-seq data, where available [6, 7] or databases collated from multiple sources, e.g. Cell Marker [32] and PanglaoDB [33]. For each sample set, Spearman correlation coefficients were generated between the TPM values of all shortlisted candidate *Ref.T*., and three were selected for each cell type (Table S1 Tab A [column A-B]), based on the following criteria: (i) A high corr. between *Ref.T*. within each cell type panel (Table S1 Tab A), indicating *cell type co-expression*: e.g., pituitary somatotrope cells [*GH1*, *GHRHR*, CD79B mean corr. ± STD: 0.84 ± 0.02] (Fig. 1A.iii and Table S1). (ii) A low corr. between *Ref.T*. across the different cell type panels (Table S1, Tab A), indicating *cell type* specificity: e.g., Somatotrope cells [*GH1*, *GHRHR*, CD79B max mean corr. with *Ref.T* for other cell types ± STD: 0.19 ± 0.03] (Fig. 1A.iii and Table S1).

#### Using reference transcript analysis to identify cell type-enriched genes

For each sample set, correlation coefficients (corr.) between each of the selected *Ref.T*. and all other sequenced protein coding genes were calculated. Whilst the cell type proportion in each sample varies due to biological and sampling variability, ratios remain relatively consistent between constitutively expressed cell-enriched genes, which principally reflect enrichment in cell types rather than regulatory networks [34]. Thus, a high corr. of a given transcript with all *Ref.T*. in *only one* cell type panel is consistent with enrichment in that cell type (see illustrative example in Fig. 1A.iv and Figure S1 for method overview). Cell type enriched genes were provisionally defined as those with: (i) minimum mean corr. >0.5 with the *Ref.T*. panel representing the cell type (all adjusted *P*-value < 0.001) and (ii) a *differential correlation* between this value and the maximum mean corr. with any other *Ref.T*. panel >0.15. The latter criteria excluded genes that were potentially co-enriched in more than one cell type, as we previously demonstrated [26]. In the case of the pituitary gland, we also performed an additional analysis where somatotropes, lactotropes and thyrotropes - three related cell types from a POU1F1-lineage [35] - were exempt from each other for differential correlation exclusion, to identify potentially co-enriched genes between these cell types (Table S1, Tab B, Column AA).

#### Unsupervised weighted network correlation analysis (WGCNA) supports Ref.T. analysis

As our analysis is based on manually selected *Ref.T*. panels, cell-type classification is subject to an input bias. Therefore, we also subjected the same GTEx RNA-seq sample sets to weighted gene correlation network analysis (WGCNA) [36] an unbiased method that does not require any manual input or marker gene selection. WGCNA generates corr. coefficients between all transcripts and subsequently clusters them into related groups, based on expression similarity (see methods for further details). In each sample set, *Ref.T*. belonging to the same cell type panel were found in the same WGCNA cluster, or clusters on the same branch or neighbouring cleaves e.g. for pituitary gland, somatotropes (cluster 55 and 38) or gonadotropes (Cluster 8) (Figure S1 G.i [grey and blue shaded boxes, respectively], Table S1 [Tab B Column W]). Overall, the majority of genes we predicted to be cell type enriched, according to the criteria above, clustered into the same WGCNA group as the corresponding *Ref.T*. or into neighbouring cleaves/related branches (Figure S1 G and S2, Table S1 [Tab B-E]), consistent with our classifications. In the small minority of cell types where this was *not* the case (6/47 cell types) the mean correlation threshold with the *Ref.T*. panel was increased from 0.50 until >50% of genes were found in neighbouring cleaves/related branches. Although our previous work showed that *Ref.T*. based analysis had a higher sensitivity than WGNCA for cell type gene enrichment analysis [25], this additional criterion was used to minimise potential false positives. All correlations and WGCNA classifications can be found in Table S1 Tabs B-E.

#### Gene ontology and protein-protein network analysis reveal cell type gene connections

Following the application of the selection criteria described above, genes classified as enriched in one cell type separated well from others analysed in the same tissue (see Fig. 1B.i-F.i for endocrine cell types in pituitary gland). Gene ontology analysis [37, 38] of the predicted cell type enriched genes revealed significant enrichment of terms linked to known functions within the tissue (Fig. 1B.ii-F.ii). For example, enriched genes in somatotropes (Fig. 1B.ii) included those linked with growth hormone receptor signalling and regulation of peptide secretion, in lactotropes (Fig. 1C.ii) included those linked to synaptic signalling and regulation of exocytosis and in gonadotropes (Fig. 1D.ii) included those linked to sex determination and gonad development. STRING protein-protein interaction analysis [39] revealed significant interactions between the proteins encoded by the genes enriched in each respective cell type (Fig. 1B.iii-F.iii).

#### Tissue specialised cell types have the highest number of enriched genes

**Fig. 2:**
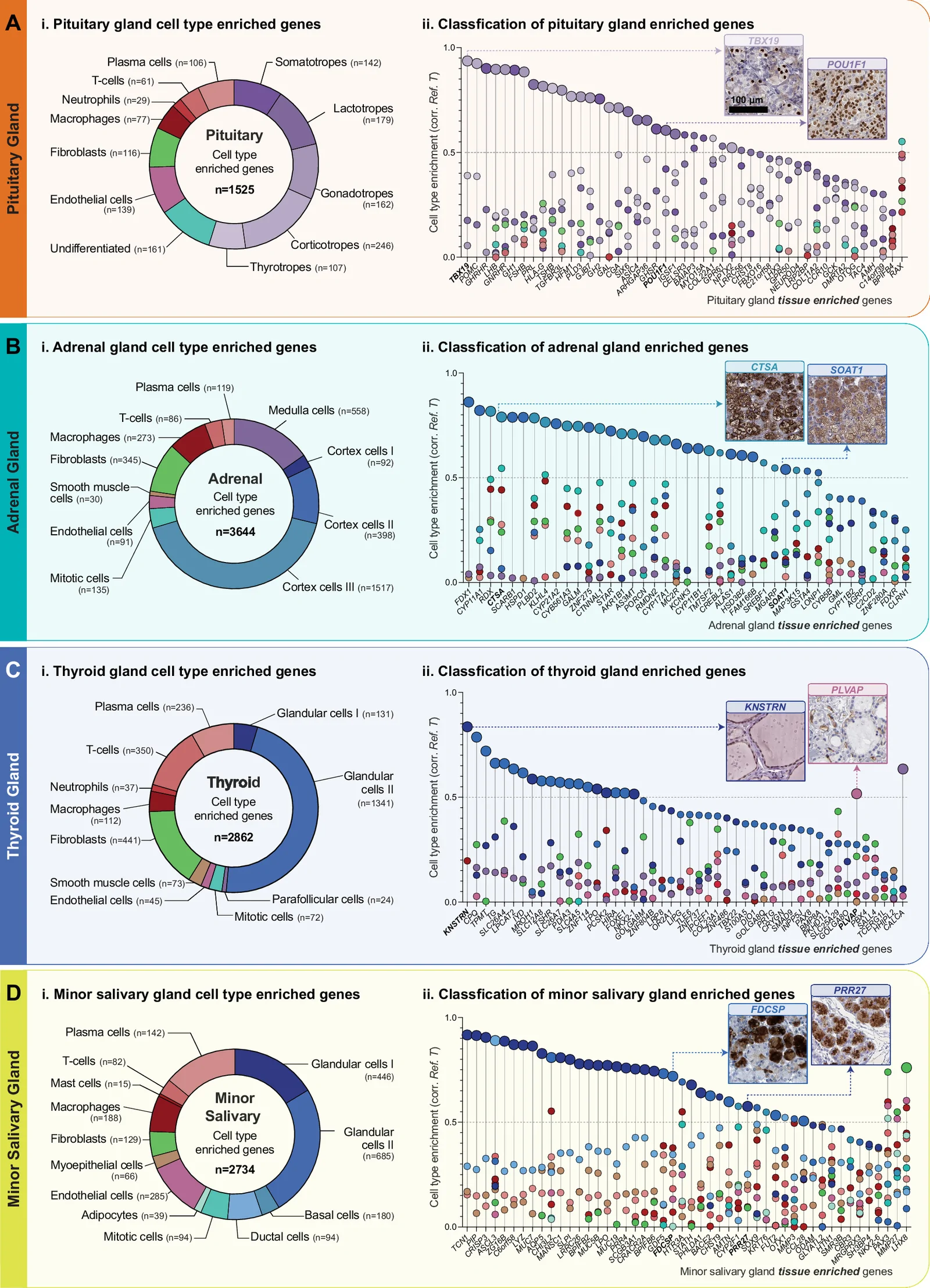
Overview of cell type enriched gene profiles across human pituitary, adrenal, thyroid and minor salivary glands. Bulk RNA-seq datasets were retrieved from GTEx V10 and cell type enriched gene predictions made using integrative correlation analysis for human: (**A**) pituitary, (**B**) adrenal, (**C**) thyroid and (**D**) minor salivary glands. (**i**) Circular plots show profiled cell type and corresponding number of predicted enriched genes (ii) cell type enrichment scores (mean correlation with the corresponding *Ref.T*) for tissue enriched genes (i.e. those expressed selectively in this tissue vs. other tissue types). Large circles indicate that the full criteria for prediction as cell type specific was fulfilled (see methods)

The total number of genes with predicted cell type enrichment within each glandular tissue ranged from 1525 in the pituitary gland (Fig. 2A.i) to 3644 in the adrenal gland (Fig. 2B.i). In all cases, tissue specialised cell types (as opposed to “core” cell types found in all or most tissues, such as endothelial cells or immune cell types) had the highest overall number of enriched genes: endocrine cell types (somatotropes, lactotropes, gonadotropes, corticotropes and thyrotropes) in the pituitary gland (836/1525 [55%]) (Fig. 2A.i), cortex cells (2007/3644 [55%]) and medulla cells (558/3644 [15%]) in the adrenal gland (Fig. 2B.i), glandular cells in the thyroid (1472/2860 [51%]) (Fig. 2C.i) and glandular cells in the minor salivary gland (1131/2734 [41%]) (Fig. 2D.i). The high number of these specialised cell type enriched genes reflect the complexity of their roles in organ specific functions.

#### Ref. T. analysis can resolve cell type source of tissue enriched genes

We collated a list of genes that were identified as tissue-enriched (i.e. with higher expression in the respective tissue vs. other tissue types) within the pituitary gland (Fig. 2A.ii), adrenal gland (Fig. 2C.ii), thyroid (Fig. 2C.ii), or minor salivary gland (Fig. 2D.ii), in both the Human Protein Atlas (HPA) [29] and GTEx [28] datasets collated in the Harminozome database [40]. The majority of pituitary enriched genes had the highest correlation values with ref. transcripts representing one or more of the five endocrine cell types (Fig. 2A.ii, enlarged circles indicate classification as cell type enriched in our analysis). Cortex cells were the predicted source of the majority of adrenal gland enriched genes (Fig. 2B.ii). Whilst most thyroid enriched genes were classified as predominantly expressed in glandular cells (Fig. 2C.ii), *PLVAP* and *CALCA* were predicted to be enriched in EC and parafollicular cells, respectively. The majority of the salivary gland enriched genes were predominantly expressed in glandular cells (Fig. 2D.ii). *LHX8* was the only salivary gland enriched gene that was classified as enriched in a non-glandular cell type with predicted enriched expression in fibroblasts. Thus, in all cases, tissue enriched genes were typically classified by our analysis as enriched in tissue specialised cell types.

#### Single cell RNA-seq is consistent with Ref.T. analysis

Compared with many other tissues, the pituitary, adrenal, thyroid and minor salivary glands are underrepresented in existing scRNA-seq studies, including large multi-tissue datasets. Thus, it was challenging to perform a comprehensive verification of our predicted cell type enriched gene classifications. However, we were able to source data from the analysis of healthy control/unaffected pituitary gland tissue from studies of pituitary neuroendocrine tumours by Zhang. Q *et al*., and sporadic pituitary adenomas by Asuzu *et al*. [41, 42], respectively, thyroid gland by Hong *et al*. [43], adrenal gland by Iwahashi *et al*., Altieri *et al*., and Huang *et al*. [44–46], and minor salivary gland from Horeth *et al*. [47], and the Human Oral and Craniofacial Cell Atlas [48–50]. Where available, we also compared our results with data from the Human Cell Landscape (HCL) database [7, 51] (thyroid and adrenal gland only) and from the Tabula Sapiens consortium [6] (minor salivary gland only). None of the scRNA-seq datasets contained all cell types we profiled, and thyroid parafollicular cells (also called C cells) were absent from all, so it was not possible to compare data for this population. As is typical, cell classification and terminology differed between studies (see Table S1 [Tab F] for nomenclature used and comparisons made).

To compare cell profiles across all datasets, we calculated the significance of overlap using a hypergeometric test for pituitary gland (Figure S3A.i), adrenal gland (Figure S3B.i), salivary gland (Figure S3C.i) and thyroid gland (Figure S3D.i). In all cases, genes predicted as cell-type enriched in our study were over-represented in the enriched genes in the corresponding cell types in the scRNA-seq studies (as classified in the respective studies themselves, or, if not provided, using fold-change vs. others threshold of ±0.25 [adjusted *p*-value < 0.001]). This overlap was comparable to, or more significant than, that between the scRNA-seq studies themselves (Figure S3A-D.i). For cell types within each tissue type, we compared the degree of overlap between the top 100 predicted cell-type-enriched genes and those similarly classified as enriched in the other datasets (Figure S3A-D.ii). For genes predicted to be cell type enriched in our analysis a high proportion were classified as such in one or more of the scRNA-seq datasets (overall for cell types in pituitary gland 54%, adrenal gland 72%, minor salivary gland 77% and thyroid 76%) (Figure S3A-D.ii, yellow plots). This replication was as good, or better than, the replication between the scRNA-seq datasets themselves.

### Comparison of cell type enriched genes across tissues reveals commonalities

**Fig. 3:**
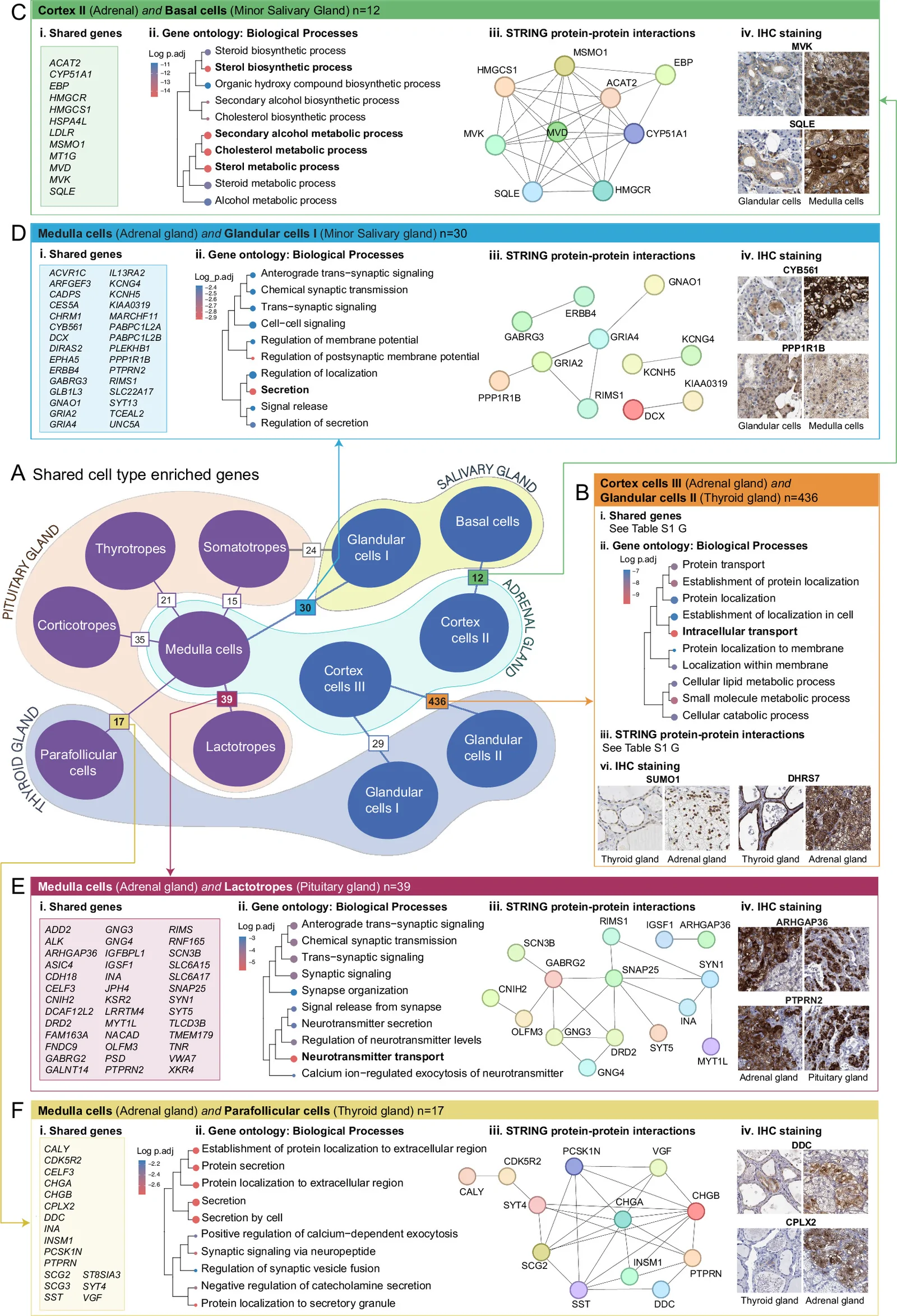
Gland specific cell types have gene enrichment signature similarities. Similarities between predicted enriched gene profiles were calculated using a hypergeometric test. **A** Bubble plot showing the number of shared enriched genes between cell types (*p* < 3.5 [Log10], group *n* > 10). For selected cell types: (**B**) cortex cells III (adrenal) and glandular cells II (thyroid), (**C**) cortex II (adrenal) and basal (minor salivary), (**D**) medulla (adrenal) and glandular I (minor salivary), (**E**) medulla (adrenal) and lactotropes (pituitary) and (**E**) medulla (adrenal) and parafollicular (thyroid), (i) a list of common enriched genes, (ii) significant gene ontology (GO) terms, (iii) evidence of interaction between the encoded proteins and (iv) examples of confirmatory tissue protein profiling (images sourced from Human Protein Atlas). See also Table S1 Tab G

While each of the glandular tissues we analysed have their own specialised functions and secreted molecules, they also share several functional and mechanistic pathways. We compared enriched gene profiles between the cell types in different tissues to find those with overlapping enrichment (summarised in Table S1 [Tab G]). We performed a hypergeometric analysis to identify cell types in the different glandular tissues with significant enriched gene profile similarities (Figure S4). As expected, common cell types found in most or all tissues analysed, such as endothelial cells, macrophages and T-cells had a highly significant overlap in enriched genes (Figure S4, marked in bold text). Various tissue specialised cell types from pituitary, minor salivary, adrenal and thyroid gland also had common enriched gene profiles (Fig. 3A summarises shared enriched gene groups where *n* = >10, *p* < 3.5 [Log10]). Adrenal cortex III cells and thyroid glandular cells II had the highest number of shared enriched genes (*n* = 436) (Fig. 3A and B). These included genes with known hormone synthesis activity, such as *FDX1* and *PGRMC1* previously reported in adrenal glands [52, 53], *SLC5A8* previously reported in the thyroid [54], and *NNT* previously reported in both [55, 56]. Others were lesser known, uncharacterised genes that, as far as we know, have no known annotated function, such as *C5orf15* and *TMEM245* (for all shared genes see Table S1 [Tab G]). Enriched GO terms for these genes included intracellular transport, protein transport and localisation, and small molecule metabolic processes (Fig. 3B.ii), and 227/436 (52.1%) had been previously classified as housekeeping genes [57]. Thus, the enriched expression here could reflect the high secretion and associated metabolic demands on these cell types. Profiling of proteins encoded by *SUMO1* and *DHRS7* showed staining consistent with expression in adrenal cortex and thyroid glandular cells (Fig. 3B.iv).

12 genes were classified as cell type enriched in both adrenal cortex cells II and salivary basal cells (p value = 2.8×10^-4^) (Fig. 3A). GO analysis revealed a strong enrichment for genes involved in steroid biosynthesis and metabolism (Fig. 3C.ii) and STRING protein-protein interaction analysis revealed significant interactions between the proteins encoded by this gene set (*p* < 1.0 × 10^−16^) (Fig. 3C.iii). These included *HMGCR*, which is a rate-limiting enzyme of cholesterol synthesis [58], together with other key genes involved in this process, including *HMGCSS1, MVK, MVD* [59] (Fig. 3C.i). Profiling of proteins encoded by *MVK* and *SQLE* showed staining consistent with expression in adrenal cortex and salivary basal cells (Fig. 3C.iv). The adrenal gland has well known primary function in production of steroid hormones for secretion to the rest of the body, however this is not a well-known function of the minor salivary glands, which are normally associated with secretion of protein rich saliva and salivary enzymes. The presence of a sterol/cholesterol metabolic signature in salivary basal cells may reflect high membrane and biosynthetic demands associated with basal/progenitor epithelial biology, rather than endocrine steroidogenesis *per se*. Nevertheless, there is emerging evidence that salivary glands may produce small amounts of certain steroid hormones (e.g., corticosterone and testosterone) [60, 61] and can metabolise circulating steroid hormones locally [62]. Further study of this shared gene signature, particularly in relation to local sterol handling, epithelial renewal, and potential intracrine steroid metabolism may help clarify its functional relevance for salivary gland biology and, potentially, salivary secretions.

30 genes were classified as cell type enriched in both adrenal medulla cells and salivary glandular cells (p value = 9.5×10^-6^) (Fig. 3D.i). These included *CYB561*, a transmembrane electron carrier involved in ascorbate metabolism, which is an essential cofactor for dopamine β-hydroxylase activity in both sympathetic nerve cells and chromaffin cells in the adrenal medulla [63], *PPP1R1B*, which is involved in dopaminergic neuronal signalling pathways and several neurological disorders [64] and *CADPS*, a membrane protein that plays a role in the regulation of neurotransmitter and hormone exocytosis [65]. GO analysis revealed an enrichment for genes involved in secretion and synaptic signalling (Fig. 3D.i). STRING protein-protein interaction analysis revealed significant interactions between groups of proteins encoded by these genes (*p*-value < 0.001) (Fig. 3D.ii). Tissue profiling of proteins encoded by *CYB561* and *PPP1R1B* showed staining consistent with expression in adrenal medulla cells and salivary glandular cells (Fig. 3D.iv).

Numerous adrenal medulla cell enriched genes were also predicted as cell type enriched in various endocrine cells of the pituitary (Fig. 3A), possibly reflecting shared roles in hormonal production and secretion. Adrenal medulla cells and lactotropes shared the highest number of enriched genes (*n* = 39) (p value < 1.0×10^-16^) (Fig. 3E.i), including those with well described roles in the regulation of secretion, e.g., *SNAP25*, *SYN1* and *PTPRN2* [66–68], those reflecting a neuroendocrine identify, e.g. *INA* and *CELF3* [69] and those involved with membrane excitability, e.g., *SCN3B* and *ASIC4* [70]. Correspondingly, GO analysis revealed an enrichment for genes involved in synaptic transmission and neurotransmitter secretion (Fig. 3E.ii). STRING protein-protein interaction analysis revealed significant interactions between groups of proteins encoded by these genes (Fig. 3D.iii) (*p* = 2.98 × 10^−10^). Tissue profiling of proteins encoded by *ARHGAP36* and *PTPRN2* showed staining consistent with expression in adrenal medulla cells and lactotropes (Fig. 3D.iv).

Most of the genes classified as enriched in thyroid parafollicular cells were also classified as enriched in adrenal medulla cells (17/24 [70.1%]) (p value < 1.0×10^-16^) (Fig. 3F.i). Co-enriched genes in this list included those involved in neuroendocrine hormone production, such as *CHGA*, *DDC*, *SYT4*, *SST* and *PTPRN* [69]. GO analysis revealed an enrichment for genes involved in protein localisation and synaptic signalling via neuropeptide (Fig. 3F.ii). STRING protein-protein interaction analysis revealed significant interactions between the proteins encoded by these genes (Fig. 3F.iii) (*p*-value < 1.0×10^-16^). Tissue profiling of proteins encoded by *DDC* and *CPLX2*, showed staining consistent with expression in thyroid parafollicular and adrenal medulla cells (Fig. 3F.iv).

#### Prediction of sex-specific differences in cell type-enriched transcripts

**Fig. 4:**
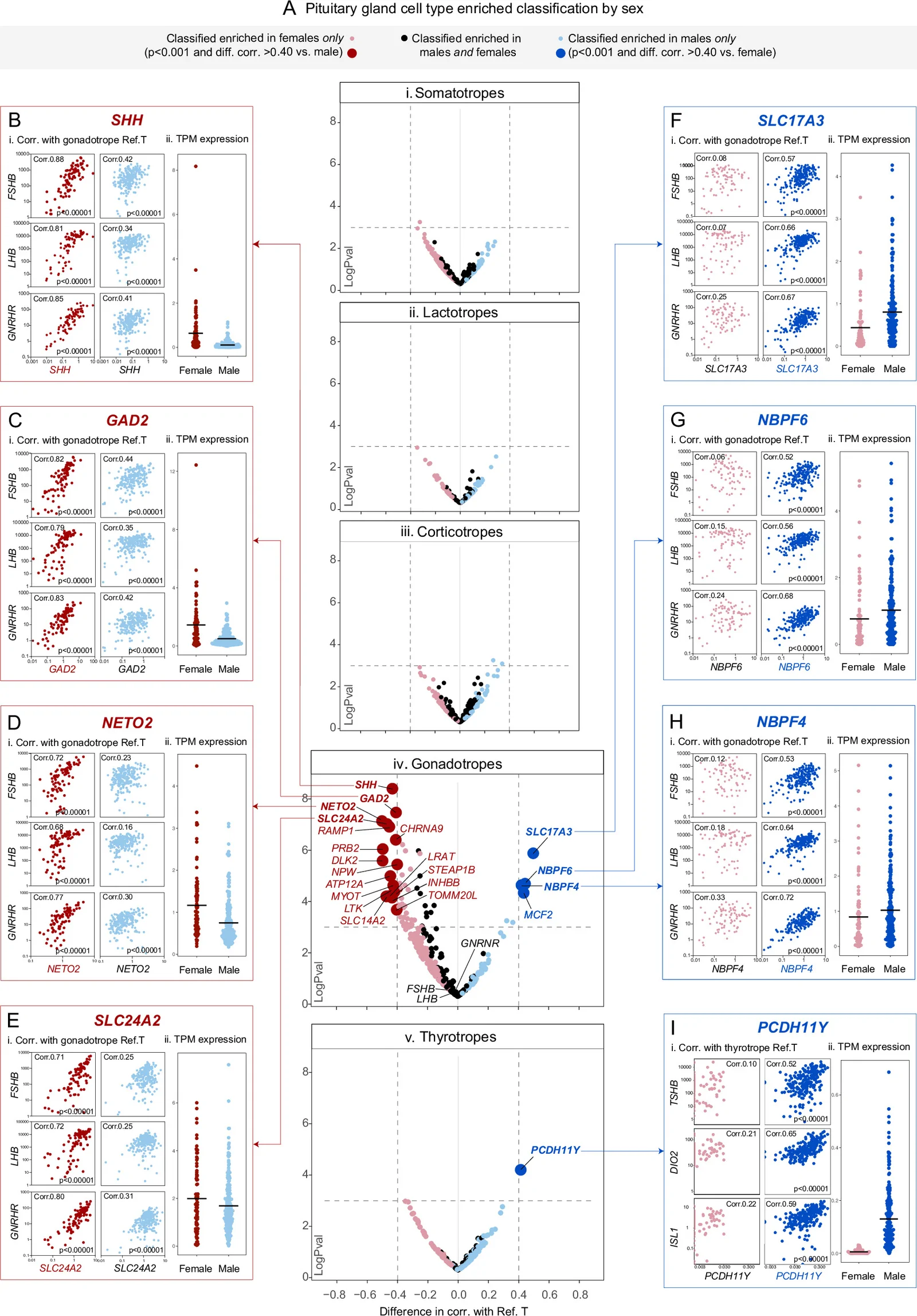
Identification of sex-specific cell-enriched genes in human pituitary gland. Pituitary gland RNA-seq data (*n* = 313 individuals) was divided into female and male subgroups (f *n* = 84, male *n* = 228) before Ref.T based analysis was performed to predict cell type-enriched genes. **A** For genes classified as enriched in: (**i**) somatotropes, (**ii**) lactotropes, (**iii**) corticotropes, (**iv**) gonadotropes and (**v**) thyrotropes in either sex, the ´*sex differential corr. score’* (difference between mean corr. with the *Ref.T*. panel in females vs. males) was plotted vs. the significance (LogPval) of difference between the respective mean correlations. For genes with higher predicted enrichment in female gonadotropes: (**B**) *SHH*, (**C**) *GAD2*, (**D**) *NETO* and (**E**) SLC24A2, or male gonadotrophs: (**F**) SLC17A3, (**G**) NBPF6, (**H**) NBPF4 and (**I**) PCDH11Y, plots show (**i**) correlation with gonadotrope *Ref.T*. in female (red/pink) or male (blue) samples, and (**ii**) TPM expression in female vs. male samples. See also Table S2 Tab A

We performed a sex-split subset analysis of the pituitary gland (female *n* = 84, male *n* = 228), thyroid gland (female *n* = 225, male *n* = 440), adrenal gland (female *n* = 110, male *n* = 184) and minor salivary glands (female *n* = 52, male *n* = 129) to compare cell type enrichment profiles in male and females. Profiles were largely comparable between sexes in the specialist cell types of the thyroid (Figure S5A; transcripts enriched in *both* males and females represented by black circles), adrenal gland (Figure S5B) and minor salivary gland (Figure S5C) (all data in Table S2). Some transcripts were classified as enriched *only* in females or males (represented by pale pink or pale blue circle symbols), however, most had relatively low differential corr. scores; indicating that they fell marginally below the designated threshold for classification as enriched in the other sex. We set a threshold for identification of male/female only enriched genes as an adjusted Fishers z-test *p*-value of <0.001 and a differential correlation score (with corresponding ref. transcripts vs. the other sex) >0.40. A small number of male-*only* cell type-enriched transcripts were identified in: thyroid glandular cells (Figure S5A, *TMSB4Y*), adrenal gland medulla and cortex cells (Figure S5B, *PCDH11Y* and *NLGN4Y*, respectively) and salivary glandular cells (Figure S5C, *TBL1Y)*. In each of these cases, transcripts were Y-linked and mRNA expression was only detected above background levels in male samples. There were no female only cell type enriched genes in the specialist cell types of the thyroid, adrenal gland or minor salivary gland (Figure S5A-C). In the pituitary gland, there were no clear sex differences between somatotrope (Fig. 4A.i), lactotroph (Fig. 4A.ii) or corticotrope (Fig. 4A.iii) gene enrichment profiles. Gonadotropes had the highest number of significant differentially-sex enriched genes, the majority of which were enriched in females only (17 vs. 4 in male) (Fig. 4A.iv).

The most significant female only enriched genes included *SHH*, a widely studied signalling molecule with many essential functions during brain and pituitary development [71]; mean corr. with gonadotrope *Ref.T* [*FSHB*, *LHB*, *GNRHR*] male=0.39 vs. female=0.82 (*p* = 5.7 × 10^−9^) (Fig. 4B.i), *GAD2*, which is responsible for the production of the major inhibitory neurotransmitter GABA; mean corr. with *Ref.T* male=0.40 vs. female=0.81 (*p* = 2.4 × 10^−8^) (Fig. 4C.i), *NETO2*, a transmembrane kainate receptor, with functions linked to modulation of synaptic transmission in neurons [72]; mean corr. with *Ref.T* male=0.23 vs. female=0.72 (*p* = 7.9 × 10^−8^) (Fig. 4D.i) and *SLC24 A2*, which is a calcium/sodium ion exchanger also expressed in several human brain regions [73, 74]; mean corr. with *Ref.T* male=0.27 vs. female=0.74 (*p* = 9.5 × 10^−8^) (Fig. 4E.i). Whilst not enriched in male gonadotropes, the latter, *SLC24 A2*, was classified as enriched in male thyrotropes in the single sex analysis (Table S2), indicating that the predominant cell type in which a given gene is enriched can vary between the sexes. In all cases, mean TPM expression of these female-only gonadotroph enriched genes was higher in female vs. male samples (Fig. 4B-E.ii). Other notable female-only gonadotrope enriched genes included *RAMP1* (Fig. 4A.iv), which is responsible for the modulation of calcitonin gene-related peptide (CGRP) receptor activity, and is known to be responsive to oestrogen and progesterone sex hormones [75]; mean corr. with *Ref.T* male=0.29, female=0.75 (*p* = 1.1 × 10^−7^), *CHRNA9* (Fig. 4A), a nicotinic acetylcholine receptor subunit, with known expression in the pituitary and responsiveness to sex hormones, and links with auditory processing and alcohol and nicotine addiction [76]; mean corr. with *Ref.T* male=0.32, female=0.75 (*p* = 5.4 × 10^−7^) and *LRAT* (Fig. 4A), which is important for both male and female fertility, with expression in ovary, testis and hypothalamus shown to be highly sensitive to FSH signalling [77, 78]; mean corr. with *Ref.T* male=0.08, female=0.53 (*p* = 3.9 × 10^−5^) (data for all in Table S2 Tab A).

Male-only gonadotrope enriched genes included *SLC17A3* (Fig. 4A), an organic anion transporter important for the excretion of uric acid in the kidneys, a function well known to be affected by sex hormones, as well as transport of other small organic anions and metabolites [79, 80]; mean corr. with *Ref.T* male=0.63 vs. female=0.13 (*p* = 1.3 × 10^−6^) (Fig. 4F.i). The closely related transporter *SLC17A4* has recently been shown to transport the thyroxine (T4) hormone [81]. SNPs associated with *SLC17A3* are linked to urid acid secretion in females but not in males, indicating a sex differentiated functionality [82]. Other significant male-only gonadotrope enriched genes included *NBPF6* (Fig. 4A); mean corr. with *Ref.T* male=0.59 vs. female=0.15 (*p* = 2.0 × 10^−5^) (Fig. 4G.i) and *NBPF4* (Fig. 4A); mean corr. with *Ref.T* male=0.63 vs. female=0.21 (*p* = 2.3 × 10^−5^) (Fig. 4H.i), both uncharacterised members of the neuroblastoma breaking point family proteins, functionally uncharacterised transcription factors with links to neuroblastoma and several other brain conditions [83], and *MCF2* (Fig. 4A) an X-linked guanine nucleotide exchange factor with sex-linked associations to Alzheimer’s development and autism [84]; mean corr. with *Ref.T* male=0.56 vs. female=0.12 (*p* = 4.6 × 10^−5^). In all cases, mean TPM expression of these genes was higher in male vs. female samples (Fig. 4F-H.ii).

*PCDH11Y*, was classified as a male-only thyrotrope enriched gene; mean corr. with *Ref.T* male: 0.59, female: 0.17 (*p* = 6.3 × 10^−6^) (Fig. 4I.i). As expected with a Y-linked gene, expressed was only detected in male samples, albeit at very low levels (Fig. 4I.ii). There were no female-only thyrotrope enriched genes. There were no other sex-specifically enriched genes in any other cell type profiled in the pituitary (Figure S5D).

## Discussion

Here we have presented a genome wide cell type transcriptome database for the human pituitary, adrenal, thyroid and minor salivary glands. Our method is based on the analysis of unfractionated bulk RNA-seq transcriptomic data [24–27], which circumvents some of the challenges associated with single cell RNA-seq, such as induction of transcriptional artifacts [8–11], difficulties with the analysis of fragile cell types and rare transcripts [9, 12, 13], and limited read depth [14–17, 20]. Also, in contrast to typical scRNA-seq-based analysis, we analyse a high number of biological replicates [21–23], allowing for well powered subgroup comparisons e.g., female vs. male. All data can be found in the Tissue Cell Type section of the Human Protein Atlas (HPA) (proteinatlas.org/humanproteome/single+cell/ tissue+cell+type).

The adrenal, pituitary and thyroid are all endocrine glands, which secrete essential hormones with body-wide effects into the bloodstream. The salivary glands are exocrine glands, secreting saliva into the oral cavity to lubricate and assist digestion. Across all four tissues, specialised cell types within each gland showed the largest number of cell type enriched genes, reflecting their functional diversity and roles. Genes that most strongly defined tissue identity (i.e., were expressed at higher levels in a given tissue compared with others) were largely those enriched in specialised resident cell types, e.g., lactotropes and corticotropes in the pituitary, or glandular cells in the thyroid, consistent with tissue-enriched signals reflecting the abundance and transcriptional programs of key cell populations [34].

We observed commonalities in cell type enrichment profiles between glands. Adrenal cortex cells and thyroid glandular cells shared over 400 enriched genes, despite producing very different endocrine hormones. The adrenal cortex is the highest-output endocrine tissue, producing cortisol in the order of tens of milligrams per day, along with other steroids [4, 85, 86]. The thyroid gland produces T4 and T3 hormones continuously [4, 5] in relatively large amounts (approximately 100μg and 29 μg daily, respectively) [87], which support systemic metabolism [5]. Thus, both glands are particularly high-capacity endocrine tissues when compared to others, such as the pituitary, which produce hormones in much smaller quantities [88]. Thus, shared genes between adrenal cortex and thyroid glandular cells could reflect the heavy demands placed on the secretory, biosynthetic and metabolic pathways required for high-capacity hormone production. Consistent with this, over 50% the genes predicted to be enriched in both were related to such ´housekeeping´ processes [57]. Thus, compared to other cells, highly metabolically or transcriptionally active cell types can express elevated levels of ´universally expressed´ genes, e.g., ribosomal or mitochondrial genes [89]. This effect can be especially pronounced in cancer, where so called “hyper transcription” can mask genes with lower expression levels and obscure cells with overall lower transcription levels [90].

The majority of genes predicted to be enriched in thyroid parafollicular cells were also predicted to be enriched in adrenal medulla cells, suggesting shared biological features; both are neuroendocrine cell types with a neural crest developmental origin [91]. Thyroid parafollicular cells are primarily known for secreting the peptide hormone calcitonin (encoded by *CALCA*), which contributes to calcium homeostasis by inhibiting osteoclast function and lowering circulating calcium concentrations [92]. In contrast, adrenal medulla cells secrete catecholamines (adrenaline and noradrenaline) that mediate the classic “fight-or-flight” response [4]. Despite these distinct hormonal outputs, both cell types rely on regulated calcium-dependent exocytosis for stimulus secretion coupling [93], and calcium/exocytosis-related gene ontology terms were over-represented among the shared enriched genes. These shared features are likely clinically relevant in multiple endocrine neoplasia type 2 (MEN2), which is caused by inherited pathogenic variants in the RET gene [94]. MEN2 is associated with a very high risk of medullary thyroid carcinoma arising from parafollicular cells, with pheochromocytoma arising from the adrenal medulla in approximately 50% of cases [94]. The co-enriched genes identified here may provide insight into shared neuroendocrine programs and mechanisms relevant to MEN2 tumorigenesis. Notably, one shared enriched gene, *DDC* (L-DOPA decarboxylase), which participates in catecholamine biosynthesis [62], has been reported as upregulated in medullary thyroid carcinoma [95].

All pituitary secretory cell types profiled shared some enriched genes with adrenal medulla cells. GO analysis showed convergence on processes related to neurotransmitter/vesicle transport and regulated secretion. Unlike the adrenal medulla, which is directly innervated via the spinal cord [4], the anterior pituitary is regulated primarily through the hypothalamo-hypophyseal portal circulation, which delivers hypothalamic releasing and inhibiting factors to pituitary endocrine cells [3, 71]. These hypothalamic neurosecretory neurons can generate action potentials [96], and modulate secretion [96–98]. Despite these differences in control, the central machinery controlling electrical excitability and stimulus–secretion coupling is strongly conserved [99]. Some of the co-enriched genes identified here may reflect shared excitable-secretory mechanisms and may have currently unknown roles in these processes.

In our analysis, predicted sex differences were most apparent in the pituitary gland, specifically in gonadotropes, which showed a comparatively high number of female-only cell type enriched genes. This is biologically plausible, as gonadotrope identity and activity is tightly coupled to sex-steroid feedback and reproductive-axis dynamics [100]. In contrast, the genes that define major cell types in the adrenal gland, thyroid and minor salivary glands predominantly reflect core, conserved functional programs that are largely shared across sexes [101]. Sonic hedgehog (*SHH*) showed the most significant difference between sexes in the pituitary, being gonadotrope enriched in females only. SHH is an essential developmental signalling molecule, particularly for brain and pituitary patterning, but previous studies linking SHH to pituitary biology have primarily focussed on its expression in corticotropes, rather than gonadotropes [71]. However, recent studies align with our findings, with *SHH* reported as enriched in chicken gonadotropes [102] and murine gonadotrope precursors [103]. Higher levels of *SHH* were found in female pituitary samples compared to male samples and one could speculate that, as female pituitary tissue undergoes dynamic adaptation across reproductive states, this SHH signal may reflect an increased plasticity/adaptability of gonadotrope transcriptional states in females. Although, to our knowledge, direct clinical implications of sex-differential *SHH* expression in gonadotropes has not been reported, *SHH* sex-based associations have been reported in other settings, e.g., SHH-subgroup medulloblastoma exhibits sex differences in some cohorts, with females reportedly having worse survival rates than males [104]. In the pituitary, Hedgehog/GLI pathway activity has been reported in adenoma biology [105, 106], but sex-stratified analyses remain limited. The most significant differentially male-only enriched gene was also in gonadotropes; *SLC17A3*, an organic anion transporter [101, 102]. Male pituitary samples had higher levels of *SLC17A3* compared to females [101], consistent with a specifically elevated expression in male gonadotropes. Male and female gonadotrope output is organized differently (tonic vs cycle/surge modes), and gonadotrope biosynthetic programs are strongly shaped by gonadotropin-releasing hormone pulse dynamics [107]. Pituitary secretion is tightly coupled to ion-channel activity/Ca²⁺ signalling and one could speculate that sex-linked enrichment of transporters, such as *SLC17A3*, could reflect differences in gonadotrope secretory state or cellular metabolite handling - although a direct role for *SLC17A3* in pituitary hormone secretion has not yet been established.

### Limitations of the study

We do not profile specific cell subtypes; it is conceptually possible to do so, but a lack of consensus regarding cell-subtype identity presents challenges. For example, in human pituitary scRNA-seq studies, gonadotropes can show transcriptional heterogeneity [108], and are frequently classified into subsets, most often along a luteinizing (LH) versus follicle-stimulating hormone (FSH) biased axis, e.g., [109], although this likely represents a functional state, rather than an actual cell subtype [110]. Thus, selection of subtype *Ref*.*T*.s required for input into our analysis model, or interpretation of WGCNA, is challenging. Thus, while our classification informs about cell-type restricted expression, it does not discriminate between genes that are expressed by all cells of a given type and those expressed only in a given sub-population. Genes may be co-enriched between related cell types within the same tissue, such as the POU1F1-lineage somatotrope, lactotrope and thyrotrope cells in the pituitary [35]. However, our study focussed on genes enriched in individual cell types and does not extensively profile such co-enriched genes. Some cell types are not profiled in our analysis, due to a lack of good cell-type-specific markers as suitable *Ref*.*T*.s. Thus, some of our predicted cell type enriched genes may also be expressed in other non-profiled cell types, a limitation that also applies to scRNA-seq tissue datasets, which frequently lack data for some constituent cell types e.g., [6]. Gene expression can be modified by genetic, epigenetic, environmental or physiological factors, such as age [111, 112]. Such genes may not correlate with the *Ref*.*T*.s, due to a variation in expression that is independent of cell-type proportions, leading to false negatives in our analysis, however it has been shown that cell type composition is the dominant cause of gene variability in bulk RNA-seq data [34]. Male samples are overrepresented in GTEx datasets, compared to female ones, which could skew the analysis. However, we also performed a sex-subset based analysis and subsequent comparison, using a Fishers z-score statistical test that takes sample size into account. We used high thresholds for classification of genes as cell type enriched, likely leading to the incorrect exclusion of some. For example, *S1PR1 and CD93* are endothelial enriched genes across tissue beds [24], but fall below the threshold for classification in the thyroid and adrenal gland in this study, respectively. However, in both cases, the enrichment score is highest in endothelial cells; thus, our classifications are intended only as a guide, and the reader should consider the data on a gene-by-gene basis.

## Materials and methods

### Experimental model and subject details

Bulk RNA-seq data analysed in this study was obtained from the Genotype-Tissue Expression (GTEx) Project (https://gtexportal.org) [30] V10 release, accessed on 2024-11-18 (dbGaP Accession phs000424.v10.p2). Biospecimen collection sites were required to obtain written informed consent from living surgery donors or written/recorded verbal authorization from the next-of-kin for deceased donor participation. Protein coding transcript types were categorised according to GENCODE v39 [107], to match with the build used by GTEx V10 (these included genes classified as “*IG_gene*” and “*TCR_gene*”). Human tissue protein profiling was performed in house as part of the Human Protein Atlas (HPA) project [29, 113, 114] (www.proteinatlas.org). Tissue samples were obtained from the Department of Pathology, Uppsala University Hospital, Sweden, as a part of the Uppsala Biobank. All tissue samples were anonymised and processed in accordance with Swedish laws and regulations, and according to approval from the Uppsala Ethical Review Board [7].

## Method details

### Tissue profiling: human tissue analysis

Immunohistochemistry (IHC) images were obtained from the Human Protein Atlas (HPA). Tissue sections were stained, as previously described [29, 113]. Briefly, formalin fixed and paraffin embedded tissue samples were sectioned, de-paraffinized in xylene, hydrated in graded alcohols and blocked for endogenous peroxidase in 0.3% hydrogen peroxide diluted in 95% ethanol. For antigen retrieval, a Decloaking chamber® (Biocare Medical, CA) was used. Slides were boiled in Citrate buffer®, pH6 (Lab Vision, CA). Primary antibodies and a dextran polymer visualization system (UltraVision LP HRP polymer®, Lab Vision) were incubated for 30 min each at room temperature and slides were developed for 10 minutes using Diaminobenzidine (Lab Vision) as the chromogen. Slides were counterstained in Mayers hematoxylin (Histolab) and scanned using Scanscope XT (Aperio). Primary antibodies are as follows: FDCSP (Origene, #2062), SUMO1 (Santa Cruz, #sc-5308) PPP1R1B (Cell Signalling, #2302). Other antibodies all from Atlas Antibodies: ARHGAP3 (HPA002064), CPLX2 (HPA071191), CTSA (HPA072487), CYB561 (HPA014753), DDC (HPA017742), DHRS7 (HPA078401), KNSTRN (HPA042027), MVK (HPA016961), PLVAP (HPA002279), POU1F1 (HPA050624), PRR27 (HPA036151), PTPRN2 (HPA007255), SOAT1 (HPA047171), SQLE (HPA020762), TBX19 (HPA072686). All IHC images are available on the Human Protein Atlas website (https://www.proteinatlas.org/).

## Quantification and statistical analysis

### Reference transcript based correlation analysis

The analysis method used to identify cell type enriched genes from bulk RNA-seq tissue data was based on our previously described model [27–29]. For each tissue, pairwise Spearman correlation coefficients were calculated between reference transcripts (*Ref.T*) selected as proxy markers for the constituent cell types (See Table S1 A) and all other sequenced genes. Transcripts with a TPM value < 0.1 in more than 50% of samples were excluded from analysis (but are still included in data tables). Correlation coefficients were calculated in R using the *corr.test* function from the *psych* package (v 1.8.4), together with False Discovery Rate (FDR) adjusted *p*-values (using Bonferroni correction) and raw *p*-values. A minimum correlation of 0.50 (FDR < 0.0001) was required for inclusion as cell type enriched. See results section for full criteria required for transcript classification of transcripts as cell-type enriched (also Figure S1). For comparisons of cell type enriched gene profiles in females vs. males, GTEx data was divided into subsets based on the sex annotation, prior to the same analysis as described above. A Fisher’s z-score was then used to calculate the significance of differences between gene enrichment scores in cell types from male vs. female samples (i.e. correlation values with the respective *Ref.T*), which also accounted for the unequal numbers of samples in the male and female sample sets.

### Weighted correlation network (WGCNA) analysis

The R package WGCNA [34] was used to perform co-expression network analysis for gene clustering, on Deseq2 data. The analysis was performed according to recommendations in the WGCNA manual. Soft threshold powers were set according to the scale free topology index (values in Table S1 Tab A), and gene clusters were calculated using a minimum size of 15. Transcripts with too many missing values were excluded using the goodSamplesGenes() function. The remaining genes were used to cluster the samples. Clusters were annotated as related to specific cell types when known marker genes were present.

### Additional dataset analysis

We sourced data from scRNA-seq analysis of healthy control/unaffected pituitary gland tissue from studies of pituitary neuroendocrine tumours by Zhang.Q *et al*., and sporadic pituitary adenomas by Asuzu *et al*. [41, 42], respectively, thyroid gland by Hong *et al*. [43], adrenal gland by Iwahashi *et al*., Altieri *et al*., and Huang *et al*. [44–46], and minor salivary gland from Horeth *et al*. [47], and the Human Oral and Craniofacial Cell Atlas [48–50]. Where available, we also used data from the Human Cell Landscape (HCL) database [7, 51] (thyroid and adrenal gland only) and from the Tabula Sapiens consortium [6] (minor salivary gland only). As is typical, cell classification and terminology differed between studies (see Table S1 [Tab F] for nomenclature used and comparisons made). Genes were classified as cell type enriched by the criteria defined in each respective scRNA-seq study, or (if none was provided), using an adjusted *p*-value threshold of 0.001 and a fold-change vs. others threshold of ±0.25. For Tabula Sapiens and CELLxGENE datasets, scRNA-seq data was processed using Seurat package for R [110], and enriched genes were identified using the FindAllMarkers function. Criteria used for comparison of our cell type-enriched datasets with expression profiles in the independent studies are given in the relevant results sections and associated tables or figure legends.

### Cross tissue/validation dataset comparisons

The statistical significance of overlap between: (i) cell-type enriched genes in the different tissue types, or (ii) genes we classified as cell type-enriched in this study vs. those classified as such in independent scRNA-seq studies, was calculated using a hypergeometric test. Heatmap and hierarchical clustering was generated by the Heatmap.2() function in the gplots R package.

### Gene ontology and protein-protein interaction analysis

Enriched Gene Ontology (GO) terms were generated using the ClusterProfiler R package [108] on 2025-05-07, and the Bioconductor human genome annotation *org.Hs.eg.db* version 3.21.0 [109]. Protein-protein interaction analysis was carried out using the core data resource STRING (https://string-db.org/) [115].

## Supplementary information

Supplementary Figures

Supplementary tables legends

Table S1

Table S2

## Data Availability

This study analysed existing, publicly available data from Genotype-Tissue Expression (GTEx) Project (gtexportal.org) V10 release. All new analysis is included in the Supplemental Tables. Analysed data for all protein coding genes is provided on the Human Protein Atlas website: (https://www.proteinatlas.org/humanproteome/single+cell/tissue+cell+type).

## Code availability

This study analysed existing, publicly available data from Genotype-Tissue Expression (GTEx) Project (https://gtexportal.org) [30] V10 release. All original code will be deposited at GitHub and will be publicly available as of the date of publication.

## Declarations

### Data usage

We used data from Genotype-Tissue Expression (GTEx) Project (https://gtexportal.org) [28], supported by the Office of the Director of the National Institutes of Health, and by NCI, NHGRI, NHLBI, NIDA, NIMH, and NINDS.
