# Supplementary material for: A cell type transcriptome atlas for human pituitary, adrenal, thyroid and minor salivary glands
Source: BMC Genomics. 2026 Nov 5;27:922. doi: 10.1186/s12864-026-13514-z (PMC13737658; doi:10.1186/s12864-026-13514-z)
Supplement: Supplementary file 1 [file 12864_2026_13514_MOESM1_ESM.docx]

**Figure S1. Overview of the analysis methodology; related to all figures.** RNAseq data for 313 unfractionated human pituitary gland samples were retrieved from GTEx V10. (**A**) Each sequenced sample contained mixed cell types, present in differing proportions. (**B**) Cell type marker genes (´reference transcripts´ [*Ref.T.*]) were selected, based on in house tissue protein profiling and/or existing literature and datasets, as a proxy for the cell proportion within each sample (e.g., *CD79B*, *GH1* and *GHRHR* for somatotropes [ST]). (**C**) Spearman correlation coefficients (corr.) between each selected *Ref.T.* and all other sequenced protein coding transcripts (~19,000) were calculated across samples and classified as: (i) not correlated, or (ii) correlated (corr. >0.50, p-value <0.00001) with the *Ref. T*. (**D**) This process was repeated for *Ref.T.* representing all cell types, and results integrated to identify genes that (**E**) correlated predominantly with only one cell type *Ref.T* panel (´differential corr.´ to next highest >0.15), which were classified as cell type enriched e.g., *NKX2-2,* or those that (**F**) did not selectively correlate with one cell type *Ref.T* panel, e.g., *COL6A6.* (**G**) Human pituitary gland RNAseq data (n=313) for all sequenced genes was subject to (i) weighted correlation network analysis (WGCNA). Large, coloured circles indicate *Ref.T.* location on resultant dendrogram and small circles indicate related cleaves annotated as representing the same cell type. (ii) Bar plot showing the distribution of genes predicted to be cell type enriched. In cases where less than 50% of the predicated cell type enriched genes fell into the correspondingly annotated cell type group, the correlation threshold (0.5) was increased until this criterion was also reached (see final minimum correlation values next to cell type colour key). See also Figure S2.

## **Figure S2. Weighted network correlation analysis (WGCNA) for adrenal, thyroid and minor salivary gland; related to all Figures 1, 2 and S1.** Human RNAseq data from (**A**) adrenal gland (n=295), (**B**) thyroid gland (n=684) and (**C**) minor salivary gland (n=181) were subject to weighted correlation network analysis (WGCNA). Bar plot shows the distribution of predicted cell type cell type genes.

**Figure S3. A comparison of cell-type enriched genes identified in our analysis and independent scRNAseq datasets cell type profiling studies**. Comparative analysis profiled cell types in (**A**) Pituitary, (**B**) Adrenal, (**C**) Minor salivary gland and (**D**) Thyroid glands showing: (**i**) Bubble heatmaps of the significance (indicated by dot size and colour) of overlap between predicted enriched genes, as identified in the current study using integrative correlation analysis of bulk RNAseq (marked yellow), and other scRNAseq databases (see method or results section for details). The size and colour of the dot represents the p-value of the hypergeometric test used for comparison. If no dot is displayed, P > 0.05 (**ii**) Barplots showing the proportion of the top 100 cell type enriched genes in each study that were similarly classified in one or more of the other studies.

.

## **Figure S4. Predicted gene enrichment signature similarities between profiled cell types across glands.** Heatmap showing significance p-values for similarity scores between predicted cell type enriched genes, calculated using a hypergeometric test. See also Figure 3.

## **Figure S5.** **Identification of sex-specific cell-enriched transcripts in human gland tissues; Related to Figure 4.** RNAseq data for each gland was divided into female and male subgroups before Ref.T based analysis was performed to predict cell type-enriched genes. For tissue specialised cell types in (**A**) thyroid, (**B**) adrenal gland and (**C**) minor salivary gland and core cell types in (**D**) pituitary gland the ´*sex differential corr. score’* (difference between mean corr. with the *Ref.T.* panel in females vs. males) is shown vs. the significance (Log of the adjusted Fishers z-test p-value) of the difference between the respective correlations in females and males. See also Table S2.
