# Supplementary material for: A cell type transcriptome atlas for human pituitary, adrenal, thyroid and minor salivary glands
Source: BMC Genomics. 2026 Nov 5;27:922. doi: 10.1186/s12864-026-13514-z (PMC13737658; doi:10.1186/s12864-026-13514-z)
Supplement: Supplementary file 2 [file 12864_2026_13514_MOESM2_ESM.docx]

**SUPPLEMENTAL TABLE LEGENDS**

**Table S1. Reference transcript selection and gene enrichment scores for all cell types.** Tab **A:** Correlation coefficient values were calculated between selected *Ref.T.* in bulk RNAseq samples (GTEx) from (i) Pituitary gland (n=313), (ii) Adrenal Gland (n=295), (iii) Thyroid gland (n=684) or iv. Minor salivary gland (n=181). Tabs **B-E**: Correlation coefficient values were calculated between selected *Ref.T.* and all other sequenced transcripts to determine cell type enrichment profiles in Tab **B**: Pituitary gland, Tab **C**: Adrenal Gland, Tab **D**: Thyroid gland or Tab **E**: Minor salivary gland. See key for column details. *Related to all figures*. Tab **F**: Cell nomenclature and groupings used for data comparison with scRNAseq studies. *Related to Figure S3.* Tab **G**: Gene signature overlaps, list of all genes comparing cell type enrichment between all 4 tissues, showing overlapping cell type enrichment. Tab **H**: Shared enriched genes between Thyroid Glandular Cells II and Adrenal Gland Cortex Cells III, and protein-protein interaction evidence. *Related to Figure 3 and Figure S4.*

**Table S2. Sex stratified subset analysis of cell-enriched transcripts.** Correlation coefficient values were calculated between selected *Ref.T.* and all other sequenced transcripts in Tab **A**: Pituitary gland, Tab **B**: Adrenal Gland, Tab **C**: Thyroid gland or Tab **D**: Minor salivary gland, subdivided into male or female only sample sets, and calculated significance in enrichment differences. See key for column details. *Related to Figure 4 and Figure S5*.
